# KLF9, Epigenetic Silenced by DNMT1, Promotes ERK‐Mediated Ferroptosis of Osteoarthritic Chondrocytes Through Transcriptionally Regulating CYP1B1

**DOI:** 10.1111/jcmm.70375

**Published:** 2025-02-27

**Authors:** Min Lv, Yuanzhen Cai, Weikun Hou, Kan Peng, Ke Xu, Chao Lu, Wenxing Yu, Weisong Zhang, Lin Liu

**Affiliations:** ^1^ Osteonecrosis and Joint Reconstruction Ward, Honghui Hospital Xi'an Jiaotong University Xi'an Shaanxi China

**Keywords:** CYP1B1, DNMT1, ferroptosis, KLF9, osteoarthritis

## Abstract

Ferroptosis plays a crucial role in the pathogenesis of osteoarthritis (OA), and inhibition of chondrocyte ferroptosis effectively alleviates OA progression. Krüppel‐like factor 9 (KLF9) is demonstrated to be upregulated in OA, but its molecular mechanism remains unclear. The study aimed to investigate the role of KLF9 in OA progression. Primary chondrocytes were treated with IL‐1β to establish an OA cell model, and showed that KLF9 was highly expressed in IL‐1β‐incubated chondrocytes. Knockdown of KLF9 alleviated IL‐1β‐induced chondrocyte degeneration. In addition, chondrocytes treated with IL‐1β showed a decreased methylation proportion in the KLF9 gene promoter. DNA methyltransferase 1 (DNMT1) directly bound to the KLF9 promoter, and overexpression of DNMT1 inhibited KLF9 expression by promoting its promoter methylation in chondrocytes. Subsequently, KLF9 shRNA and pcDNA‐CYP1B1 were individually or altogether transfected into chondrocytes. KLF9 shRNA inhibited Cytochrome P450 1B1 (CYP1B1) expression in chondrocytes, and pcDNA‐CYP1B1 abrogated the inhibitory effect of KLF9 shRNA on IL‐1β‐induced chondrocyte ferroptosis. Moreover, Ferrostatin‐1 (Fer‐1, an inhibitor of ferroptosis) reversed the promotion of pcDNA‐CYP1B1 on IL‐1β‐induced chondrocyte ferroptosis. Finally, in vivo experiments showed that KLF9 shRNA significantly suppressed the cartilage tissue damage, ferroptosis, and the IHC scores of KLF9 and CYP1B1 in rats. In conclusion, our results suggested that KLF9, epigenetic silenced by DNMT1, promoted extracellular signal‐regulated kinase (ERK)‐mediated ferroptosis of OA chondrocytes through transcriptionally regulating CYP1B1. Thus, KLF9 is expected to be a new target for the treatment of OA.

## 1 Introduction

Osteoarthritis (OA) is a common chronic degenerative joint disease characterised by cartilage damage, which ultimately leads to cartilage degeneration, joint pain and stiffness. More than 300 million people worldwide suffer from OA, which seriously affects their daily life and work [1, 2]. Despite the extensive research conducted on the pathogenesis and therapeutic targets of OA, there remains a deficiency in effective and safe diagnostic and therapeutic interventions [3]. Therefore, in‐depth research on the pathogenesis of OA and identification of effective clinical treatment targets are crucial for the treatment of OA. Chondrocytes, as the sole resident cellular component of cartilage tissue, and their degeneration and death contribute to the progression of OA [4]. A previous study showed that chondrocyte damage, including necroptosis, apoptosis and autophagy were crucial for the occurrence and progression of OA [5]. Therefore, inhibiting chondrocyte apoptosis and necroptosis can improve the prognosis of OA and are crucial for the treatment of OA.

Ferroptosis is a novel form of cell death characterised by lipid peroxidation [6]. More and more studies are focusing on the role of ferroptosis in OA [7]. Inhibiting ferroptosis during the pathological progression of OA can promote chondrocyte proliferation and cartilage matrix synthesis [8, 9]. Furthermore, the ferroptosis inhibitor Fer‐1 has the capacity to inhibit ferroptosis stimulated by inflammatory environment in primary chondrocytes [10]. Therefore, inhibition of chondrocyte ferroptosis may serve as a therapeutic research strategy for OA.

Kruppel‐like factor 9 (KLF9), a transcription factor belonging to the Krüppel‐like family [11]. The carboxyl terminus of these protein harbours three highly conserved Cys2/His2 zinc finger domains, which can bind to promoters or enhancers and regulate the expression of their corresponding target genes [12]. Consequently, they have diverse biological functions. Previous research demonstrated that the KLF family played a pivotal role in metabolic processes within organisms [13, 14]. A bioinformatics analysis showed that KLF9 was significantly increased in OA and expected to become a novel therapeutic target for OA progression [15]. However, its regulatory mechanisms in OA have not yet been elucidated.

DNA methylation is catalysed by a group of conserved enzymes called DNA methyltransferases, which are participate in various pathophysiological processes, including tumorigenesis, inflammation and aging [16]. A recent study indicated that the development of OA is associated with epigenetic changes in many OA‐susceptible genes. DNA hypermethylation or hypomethylation leads to the activation or suppression of OA genes [17]. DNA methylation is usually catalysed by DNMT1, DNMT3A and DNMT3B [18]. DNMT1 maintains DNA methylation marks during cell division, while DNMT3A and DNMT3B catalyse de novo DNA methylation during development [19]. It has been reported that DNMT3B was crucial for maintaining cartilage homeostasis, and the loss function of DNMT3B led to cartilage catabolism [20]. High expression of DNMT3A restricts the proliferation and anti‐apoptosis of OA chondrocytes [21]. DNMT1‐mediated hypermethylation of the PPARG promoter leads to its suppression, which may be an effective target for exerting chondroprotective effects [22]. However, it is still unclear which methyltransferase acts on KLF9.

In this study, we elucidated the role of KLF9 in the progression of OA and its specific molecular mechanism by constructing an OA model in vivo and in vitro with the aim of identifying potential targets for the treatment of OA.

## 2 Materials and Methods

### 2.1 Cell Culture and Transfection

Primary human cartilage cells were purchased from Shanghai Jinyuan Biotechnology Co., LTD. Cells were cultured using DMEM medium containing 10% fetal bovine serum, 100 U/mL penicillin and 100 mg/mL streptomycin at 37°C in an incubator with 5% CO_2_. When the cells reached 80% confluence, they were digested with 2.5% trypsin and passaged. IL‐1β as a proinflammatory factor, could stimulate chondrocytes and lead to the occurrence of OA. Therefore, in this study, a concentration of 10 ng/mL of IL‐1β was used to treat cells to establish an OA cell model. KLF9 shRNA (top 5′‐CCGGGGCCCTTTCCCTGCACGCTCGAGCGTGCAGGGAAAGGGCCTTTTTG‐3′ and bottom 5′‐AATTCAAAAAGGCCCTTTCCCTGCACGCTCGAGCGTGCAGGGAAAGGGCC‐3′), pcDNA‐CYP1B1, pcDNA‐DNMT1 and their negative controls were purchased from Shanghai Hanao Biotechnology Co., LTD. All transfection reagents were used to transfect chondrocytes for 48 h using Lipofectamine 3000 (Invitrogen) transfection reagent.

### 2.2 RT‐qPCR Analysis

Total RNA was extracted from chondrocytes using 1 mL TRIzol reagent (Invitrogen, Carlsbad, CA) and lysed on ice for 3 min to ensure no contamination during the extraction process. Total RNA was reverse transcribed into cDNA using Prim‐script RT kit, and cDNA was used as template for PCR amplification under the action of DNA polymerase. SYBR‐qPCR Master Mix kit (Takara, Tokyo, Japan) was used to set the PCR system and reaction conditions. Relative gene expression was calculated using the 2^−ΔΔCT^ method. Primers used in the study includes: KLF9, forward 5′‐TACATGGACTTCGTGGCTGC‐3′, reverse 5′‐AGGGCCGTTCACCTGTATGC‐3′; CYP1B1 forward 5′‐CACCTCTGTCTTGGGCTACC‐3′ reverse 5′‐TTCGCAGGCTCATTTGGGTT‐3′; DNMT1 forward: 5′‐GGGGACGACGGGAAGACCT‐3′, reverse: 5′‐CCGGCCAATTCGGTAGGG‐3′; β‐actin, forward, 5′‐TCGTGCGTGACATTAAGGAG‐3′, reverse: 5′‐GTCAGGCAGCTCGTAGCTCT‐3′.

### 2.3 Western Blotting

Radioimmuno‐precipitation assay (RIPA) cell lysate was added to chondrocytes and lysed on ice for 15 min to extract proteins. The protein concentration was then determined by Bicinchoninic acid (BCA) kit (Beyotime, Shanghai, China), and the proteins were separated by SDS‐PVDF electrophoresis, and then transferred to Polyvinylidene fluoride (PVDF) membrane, and the membrane was rotated for about 60 min according to the protein size. After blocking with the final 5% skim milk powder for 2 h, the cells were incubated overnight at 4°C with the following primary antibodies: β‐actin antibody (1:5000, Abcam, ab8226), collagen II antibody (1:1500, Abcam, ab30674), MMP13 antibody (1:3000, Abcam, ab39012), ADAMTS‐5 antibody (1:1000, Abcam, ab41037), Aggrecan antibody (1:500, Abcam, ab3778), GPX4 antibody (1:1000, Abcam, ab125066), KLF9 antibody (1:1000, Abcam, ab313477), CYP1B1 antibody (1:1000, Abcam, ab185954), DNMT1 antibody (1:500, Abcam, ab188453), ACSL4 antibody (1:2000, Abcam, ab155282), SLC7A11 antibody (1:1000, Abcam, ab307601). Then, horseradish peroxidase (HRP) conjugated goat anti‐rabbit IgG (1:2000, Abcam, ab6721) for 1 h at 4°C. The ECL developer solution was uniformly added to the PVDF membrane, and the immunoreactive bands were visualised using a gel imaging system (Bio‐Rad, USA). ImageJ software was used to analyse the grey value of each band.

### 2.4 CCK8 Assay

Our study adopted cell counting kit‐8 (CCK‐8) assay to detect cell viability. The transfected chondrocytes were cultured at a density of 1 × 10^4^ cells/well in 96 wells. After 24 h, add 10 μL of CCK8 solution to each well and continue incubating for 3 h. Finally, applied an enzyme‐linked immunosorbent assay (ELISA) reader to detect the absorbance at a wavelength of 450 nm.

### 2.5 Propidium Iodide (PI Staining) Analysis

A single‐cell suspension of chondrocytes was inoculated into a six‐well plate at a density of 2 × 10^5^ cells/well. Cells were treated with SchA (0, 25 and 50 μmol/L) at 37°C for 24 h, washed twice with PBS, and incubated with PI (5 μg/mL) at room temperature for 15 min. Cells were subsequently washed three times with PBS and observed to emit blue and red fluorescence. Images were captured using a fluorescence microscope. Monochromatic fluorescence images were merged using Image software.

### 2.6 Determination of Malondialdehyde (MDA) and Glutathione (GSH) Contents

Cells were lysed by RIPA for 15 min, and the sample and MDA standard solution were added, mixed well, and heated to 100°C, cooled in a water bath and centrifuged at room temperature, the supernatant was aspirated into a 96‐well, and the microplate reader was set at a wavelength of 532 nm for OD measurement. After reaction with GSH at room temperature, absorbance was measured at a wavelength of 411 nm.

### 2.7 Reactive Oxygen Species (ROS) Content Detection

ROS levels were determined using the DCFH‐DA fluorescent probe. DCFH‐DA was prediluted at a ratio of 1:1000 until the concentration reached 10 μmol/L for cell treatment. Cells were incubated at 37°C for 30 min and washed three times with PBS. FITC flow cytometry was used for detection (excitation wavelength 500 nm, detection wavelength 525 nm).

### 2.8 Methylation‐Specific Polymerase Chain Reaction (MSP)

The genomic DNA of the cells was extracted using a DNA extraction kit (Beyotime, Shanghai, China), and the purified modified DNA was used for methylation‐specific polymerase chain reaction (MSP) or sulfite sequencing along with 100 ng of sulfite‐modified DNA, which included both methylation and non‐methylation specific markers. The modified DNA was used for PCR for MSP after selecting he appropriate primers. PCR conditions were as follows: denaturation at 95°C for 3 min, annealing at 55°C for 30 s, extension at 72°C for 1 min for a total of 35 cycles, and a final extension at 72°C for 10 min. Finally, the reaction products were analysed by electrophoresis on a 0.3% agarose gel and imaged using a gel electrophoresis instrument.

### 2.9 Chromatin Immunoprecipitation Assay (ChIP)

ChIP assay was analysed using the sonication ChIP Kit (Beyotime, Shanghai, China). Cells were fixed in 1% formaldehyde for 10 min at room temperature. Subsequently, glycine was added and vortexed and mixed to terminate the cross‐linking. The fragments of DNA are then processed by the cell lysate for between 200 and 1000 bp. Chromatin fragments were incubated overnight at 4°C with the following antibodies: Rabbit isotype IgG (mAb #3900, Cell Signalling Technology), DNMT1 (PA5‐77911, 1:50 dilution, ThermoFisher Science) and KLF9 (ab227920, 1:50 dilution, Abcam), CYP1B1 (AF6645, 1:50 dilution) were added to bind protein. 10% of the starting supernatant was used as input. The obtained DNA fragments were purified using a centrifugal column followed by qPCR analysis. The following primers were used: DNMT1 forward, 5′‐CCT AGC CCC AGG ATT ACA AGG −3′ and reverse, 5′‐ACT CAT CCG ATT TGG CTC TTT C′; KLF9 forward, 5′‐TAC ATG GAC TTC GTG GCT GC‐3′, reverse 5′‐AGG GCC GTT CAC CTG TAT GC‐3′; CYP1B1 forward, 5′‐TGC CTG TCA CTA TTC CTC ATG CCA‐3′, reverse 5′‐TCT GCT GGT CAG GTC CTT GTT GAT‐3′.

### 2.10 Luciferase Reporter Gene Assay

CYP1B1 overexpression vector and recombinant luciferase reporter gene vectors were co‐transfected into chondrocytes at the binding site. After 48 h, the original medium was discarded and the cells were washed twice with 500 μL of PBS buffer. Then, the cells were shaken slightly with lysis buffer (PLB) for 15 min to fully lysate the cells. Subsequently, luciferase experimental buffer II (LARII) and the mixed lysate were added into the test tube and placed in the detector to detect luciferase activity.

### 2.11 Animals

Forty male rats (weighing 180–280 g, 6–8 weeks) were placed in a confined space filled with pentobarbital sodium and euthanized by cervical dislocation after anaesthesia. Rats were randomly divided into four groups: the sham group, OA group, OA + NC shRNA group and the OA + KLF9 shRNA group. The sham group only exposed the knee joint without excising the meniscus or the ligaments. After surgery, the OA + NC shRNA group and the OA + KLF9 shRNA group of rats were respectively injected with a control vector or a KLF9 lentiviral interference vector at their knee joint cavities. The Sham and OA groups were given an equivalent amount of saline as a substitute. After 1 month of euthanizing the rats, the skin and muscle tissue of the lower limbs were removed, while the knee joint capsule was preserved. Fix the specimen with 4% paraformaldehyde for 48 h and perform subsequent decalcification. All animal experimental procedures were approved by the Ethics Committee of Xi'an Jiaotong University (No. XJTULAC‐2023‐116).

### 2.12 Safranin Solid Green Dyeing

Cartilage tissue slices were added with xylene, anhydrous ethanol, and alcohol for gradient dewaxing, and then stained in safranin staining solution for 1 h, followed by rinsing with deionised water. After further alcohol gradient decolorization, place it in a solid green dye solution for 40 s for staining. Finally, dehydrate and seal the film and observe the staining of the tissue under a microscope.

### 2.13 Statistical Analysis

All statistical analyses were performed using SPSS software 21.0. Data are expressed as mean ± SEM deviation. *t*‐test was used to evaluate the statistical difference between the two groups, and one‐way analysis of variance was used to compare the multiple groups (one‐way ANOVA), followed by Tukey's post hoc analysis. All experiments were performed at least three times, and *p* < 0.05 was considered statistically significant.

## 3 Results

### 3.1 KLF9 Level Was Elevated in IL‐1β‐Treated Primary Chondrocytes

**FIGURE 1:**
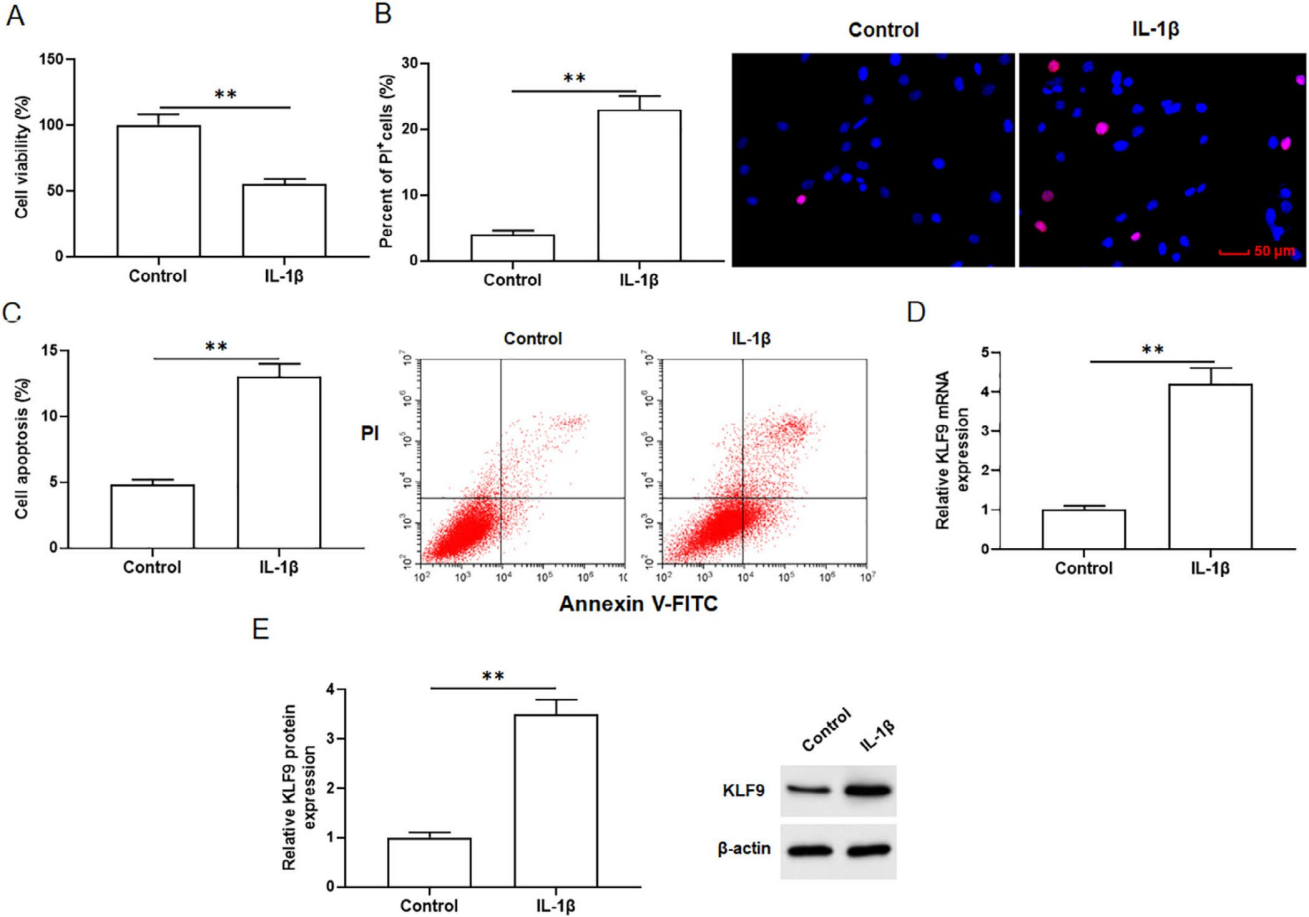
KLF9 level was elevated in IL‐1β treated primary chondrocytes. Primary chondrocytes were treated with 10 ng/mL IL‐1β for 24 h. (A) Cell viability was analysed by CCK8 assay. (B) Cell death was analysed with PI staining. (C) Cell apoptosis was analysed with Flow cytometry. KLF9 mRNA (D) and protein (E) levels were analysed by qPCR and Western blotting. All data are shown as means ± SEM. *N* = 6. Student's *t*‐test was used to calculate the differences between two groups. **p < 0.01.

Our previous research showed that the optimal concentration of IL‐1β for treating primary chondrocytes was 10 ng/mL and the optimal treatment time was 24 h [10]. Therefore, chondrocytes were treated with 10 ng/mL IL‐1β for 24 h. CCK8 assay indicated that IL‐1β treatment inhibited primary chondrocyte viability (Figure 1A). PI staining revealed that IL‐1β treatment promoted primary chondrocyte death (Figure 1B). Flow cytometry results showed that IL‐1β treatment significantly promoted chondrocyte apoptosis (Figure 1C). qPCR and Western blotting demonstrated that KLF9 level was higher in chondrocytes treated with IL‐1β than that in the control group (Figure 1D,E). These data confirmed that KLF9 was highly expressed in IL‐1β stimulated chondrocytes.

### 3.2 Knockdown of KLF9 Inhibited IL‐1β‐Induced Chondrocyte Degeneration

**FIGURE 2:**
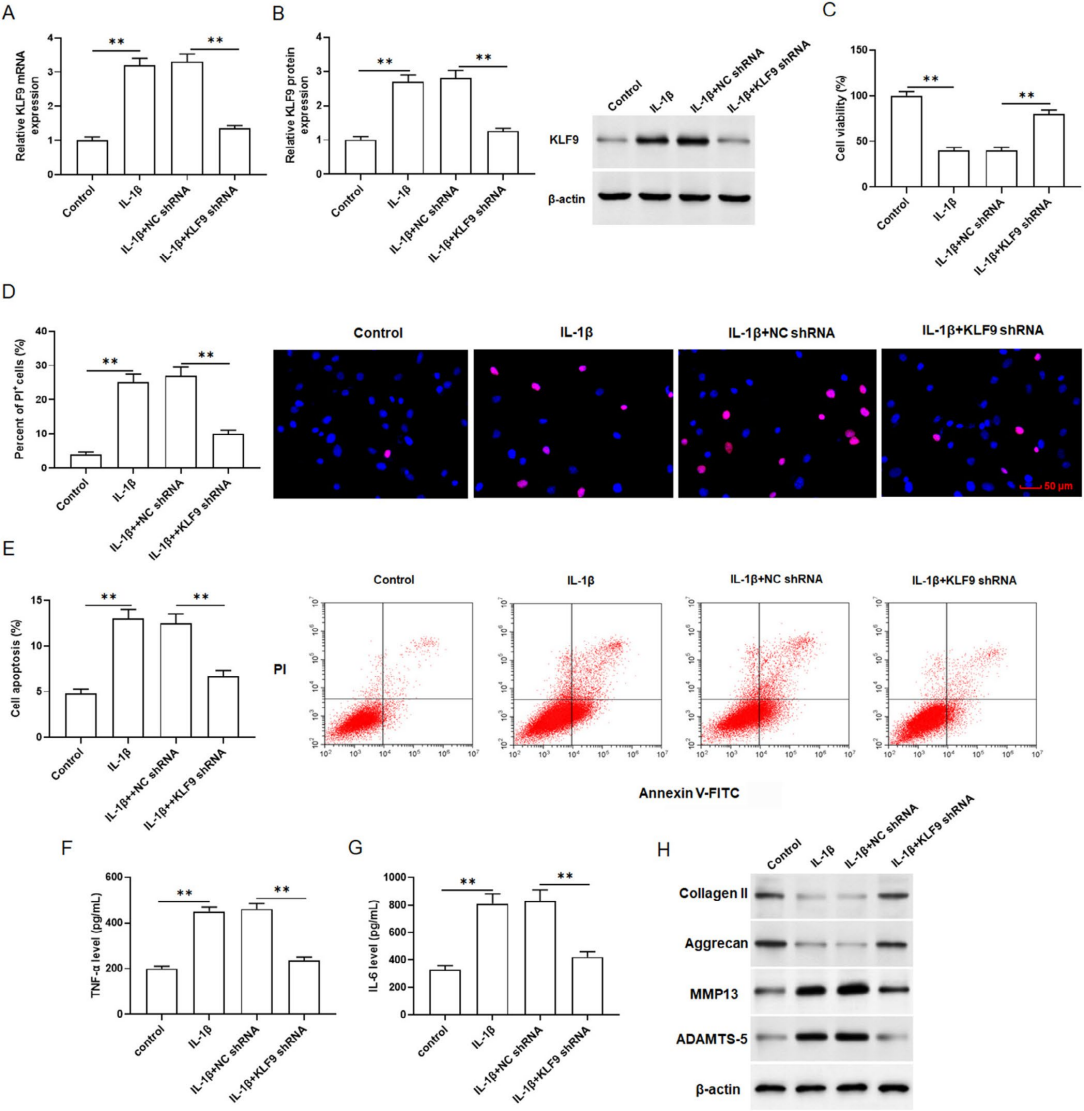
Knockdown of KLF9 inhibited IL‐1β‐induced chondrocyte degeneration. Primary chondrocytes treated with IL‐1β were transfected with KLF9 shRNA. The levels of KLF9 mRNA (A) and protein (B) were analysed by qPCR and Western blotting. (C) Cell viability was analysed by CCK8 assay. (D) Cell death was analysed with PI staining. (E) Cell apoptosis was analysed by Flow cytometry. (F, G) TNF‐α and IL‐6 secretion levels were detected by ELISA kits. (H) The protein expression of collagen II, Aggrecan, MMP13 and ADAMTS‐5 in cells were analysed by Western blotting. All data are shown as means ± SEM. *N* = 6. One way analysis of variance (ANOVA) followed by Tukey HSD test were applied for evaluating the significance among multiple groups. **p < 0.01.

To explore the role of KLF9 in OA, IL‐1β‐treated primary chondrocytes were transfected with KLF9 shRNA. qRT‐PCR and Western blotting exhibited that KLF9 shRNA inhibited the elevation of KLF9 level in IL‐1β treated chondrocytes (Figure 2A,B). CCK8 analysis suggested that downregulation of KLF9 reversed the inhibitory effect of IL‐1β on chondrocyte viability (Figure 2C). Moreover, PI staining revealed that IL‐1β treatment markedly increased chondrocyte death, while KLF9 shRNA repressed the induction of chondrocyte death by IL‐1β (Figure 2D). Flow cytometry results revealed that IL‐1β treatment significantly promoted chondrocyte apoptosis, while KLF9 shRNA repressed IL‐1β‐induced chondrocyte apoptosis (Figure 2E). In addition, TNF‐α and IL‐6 levels were significantly upregulated in IL‐1β‐incubated chondrocytes, but transfection with KLF9 shRNA effectively repressed IL‐1β‐induced chondrocyte inflammation (Figure 2F,G). Furthermore, Aggrecan and Collagen II levels were significantly decreased, while MMP13 and ADAMTS‐5 levels were significantly elevated in IL‐1β‐treated chondrocytes, while knockdown of KLF9 eliminated these changes (Figure 2H and Figure S1A–D). Collectively, these findings indicated that knockdown of KLF9 alleviated IL‐1β‐stimulated chondrocyte degeneration.

### 3.3 Overexpression of DNMT1 Inhibited KLF9 Expression

**FIGURE 3:**
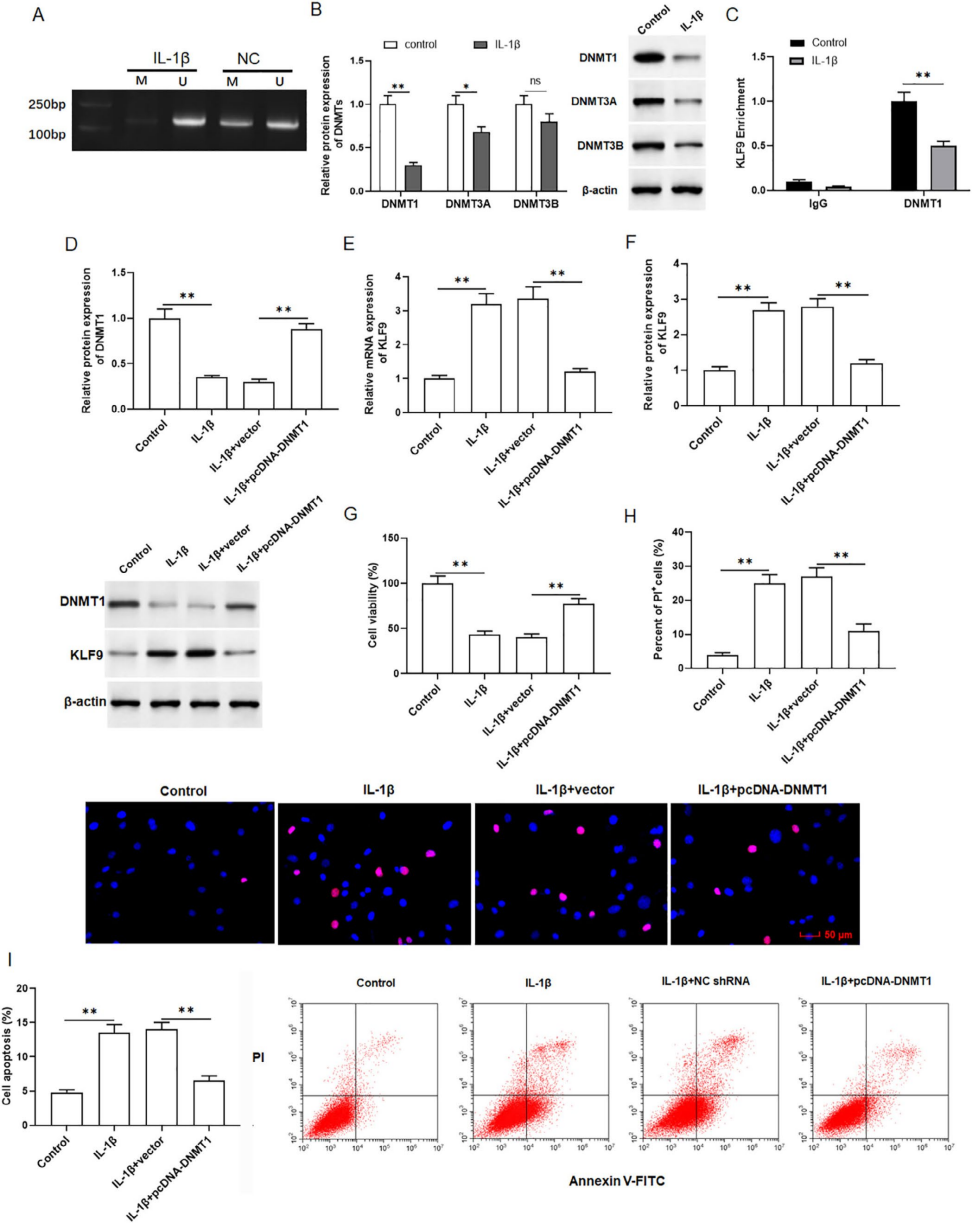
Overexpression of DNMT1 inhibited KLF9 expression. Primary chondrocytes were treated with 10 ng/mL IL‐1β for 24 h. (A) The methylation level of KLF9 was detected by MSP. (B) Western blotting was performed to assess the expression of DNMT1, DNMT3A and DNMT3B. (C) The interaction between DNMT1, DNMT3A, DNMT3B and KLF9 promoter were examined by Ch‐IP assay. The primary chondrocytes were transfected with pcDNA‐DNMT1, and then treated with 10 ng/mL of IL‐1β for 24 h. (D) Western blotting was performed to detected DNMT1 protein expression. qRT‐PCR (E) and Western blotting (F) were performed to assess KLF9 mRNA and protein expression. (G) Cell viability was analysed by CCK8 assay. (H) Cell death was analysed with PI staining. (I) Cell apoptosis was analysed with Flow cytometry. All data are shown as means ± SEM. *N* = 6. One way analysis of variance (ANOVA) followed by Tukey HSD test were applied for evaluating the significance among multiple groups. *p < 0.05, **p < 0.01.

DNA methylation is an epigenetic mechanism that involved in the development of primary OA [17]. We then further explore the role of DNA methyltransferases in OA and how KLF9 expression is regulated. First, we used IL‐1β (10 ng/mL) to incubate chondrocytes. MSP assay indicated a decreased methylation proportion in the KLF9 gene promoter in chondrocytes treated with IL‐1β (Figure 3A). We then determined which methyl transfers (DNMT1, DNMT3A and DNMT3B) were involved in the methylation of KLF9 in IL‐1β‐incubated chondrocytes. Western blotting results showed that the expression of DNMT1, DNMT3A and DNMT3B of chondrocytes in the IL‐1β group were decreased, especially DNMT1 (Figure 3B). The Ch‐IP assay results verified that DNMT1 could bind to KLF9 promoter (Figure 3C). Moreover, chondrocytes were transfected with pcDNA‐DNMT1 and treated with 10 ng/mL IL‐1β for 24 h. Western blotting showed that pcDNA‐DNMT1 elevated the level of DNMT1 in IL‐1β‐incubated chondrocytes, indicating that transfection was successful (Figure 3D). Next, we examined the influence of DNMT1 overexpression on KLF9 expression. qRT‐PCR and Western blotting results showed that pcDNA‐DNMT1 reversed the promoting effect of IL‐1β on KLF9 expression (Figure 3E,F). And overexpression of DNMT1 also rescued the decreased in viability of IL‐1β‐incubated chondrocytes (Figure 3G). The results of PI staining showed that cell death in IL‐1β‐incubated chondrocytes was notably increased, whereas overexpression of DNMT1 suppressed chondrocyte death (Figure 3H). Flow cytometry assay results showed that cell apoptosis in IL‐1β incubated chondrocytes was significantly increased, while overexpression of DNMT1 suppressed chondrocyte apoptosis (Figure 3I). Overall, these data suggested that DNMT1 increase KLF9 methylation and inhibit KLF9 expression in IL‐1β‐incubated chondrocytes.

### 3.4 Knockdown of KLF9 Inhibited IL‐1β‐Induced Ferroptosis in Chondrocytes

**FIGURE 4:**
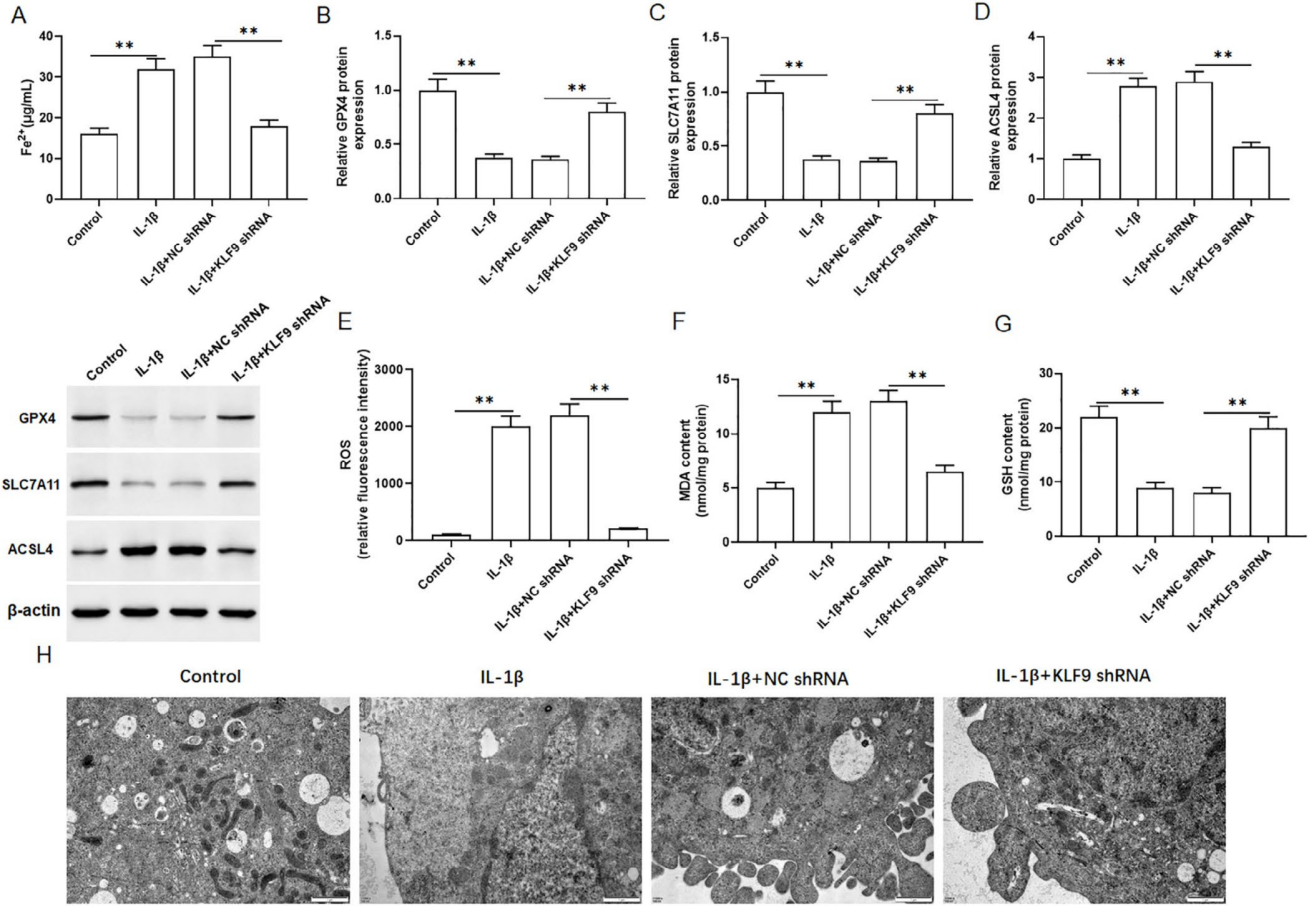
Knockdown of KLF9 inhibited IL‐1β‐induced ferroptosis in chondrocytes. Primary chondrocytes were transfected with the KLF9 shRNA, and treated with 10 ng/mL IL‐1β for 24 h. (A) The content of Fe^2+^ was detected by using kits. The protein expression of GPX4 (B), SLC7A11 (C) and ACSL4 (D) were analysed by Western blotting. The levels of ROS (E), MDA (F) and GSH (G) were detected with kits. (H) Mitochondrial morphology was observed by Transmission electron microscopy. All data are shown as means ± SEM. *N* = 6. One way analysis of variance (ANOVA) followed by Tukey HSD test were applied for evaluating the significance among multiple groups. **p < 0.01.

To determine whether KLF9 is involved in ferroptosis. The primary chondrocytes were transfected with the KLF9 shRNA, and treated with 10 ng/mL of IL‐1β for 24 h. As shown in Figure 4A, the Fe^2+^ content was increased in primary chondrocytes treated with IL‐1β, whereas KLF9 shRNA decreased the Fe^2+^ content. The results of Western blotting indicated that GPX4 (Figure 4B) and SLC7A11 levels (Figure 4C) were decreased in primary chondrocytes treated with IL‐1β, whereas KLF9 shRNA increased the levels of GPX4 and SLC7A11. Moreover, KLF9 shRNA also reversed the promoting effect of IL‐1β on ACSL4 protein expression (Figure 4D). In addition, we observed an increase in ROS and MDA content, as well as a decrease in GSH content in IL‐1β‐treated primary chondrocytes (Figure 4E–G). However, the effect of IL‐1β on these metrics in chondrocytes was reversed by KLF9 shRNA. Additionally, the ultra‐structure of the mitochondria was observed by TEM and results showed that in IL‐1β‐incubated chondrocytes, mitochondrial shrinkage, mitochondrial outer membrane rupture and reduction of mitochondrial cristae were observed, while KLF9 shRNA significantly restored these pathological damages to mitochondria (Figure 4H). These data indicated that knockdown of KLF9 suppressed IL‐1β‐induced chondrocyte ferroptosis.

### 3.5 Knockdown of KLF9 Inhibited the Expression of CYP1B1 in Osteoarthritis

**FIGURE 5:**
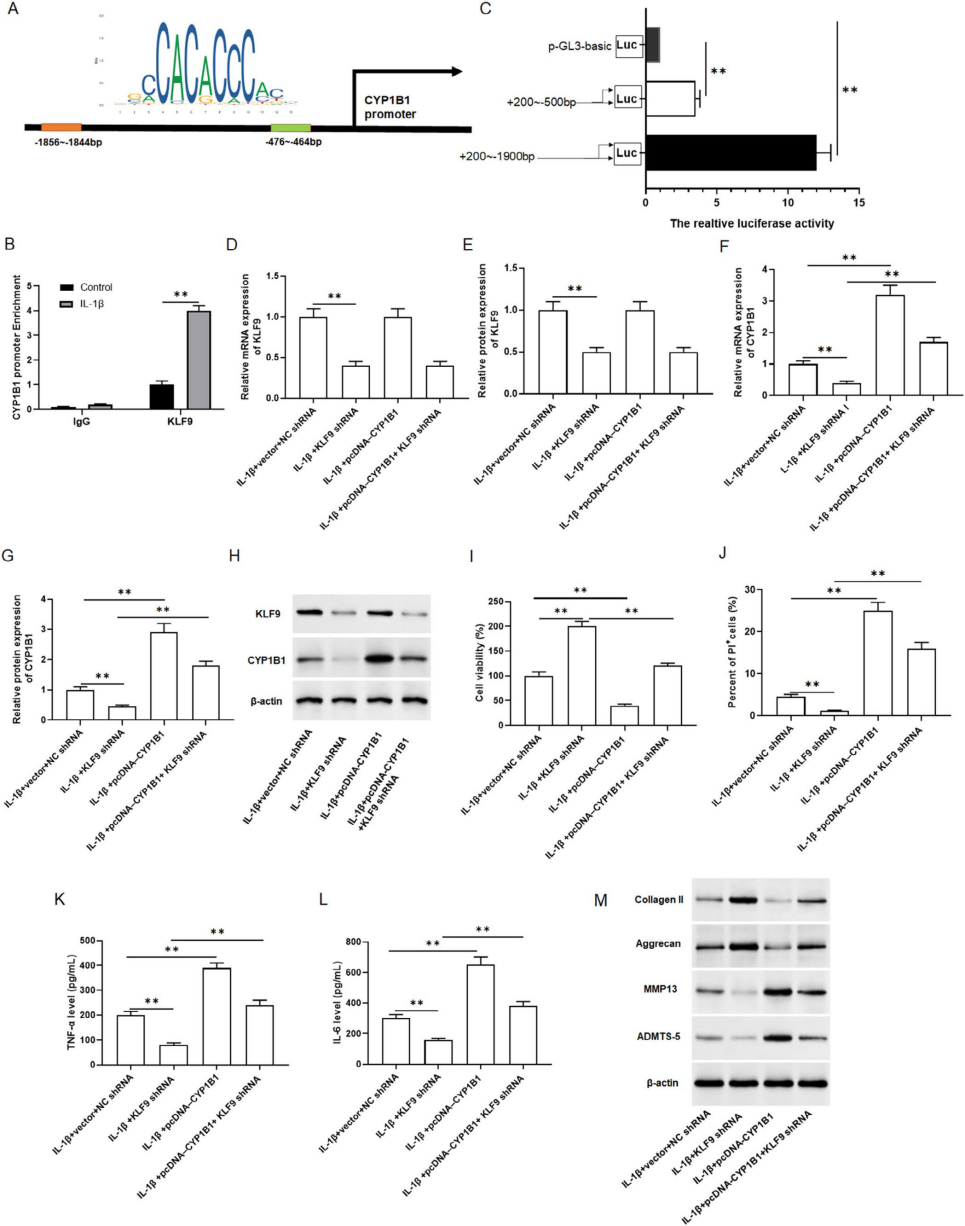
Knockdown KLF9 inhibited the expression of CYP1B1 in osteoarthritis. (A) The binding sites of KLF9 on CYP1B1 promoter was predicted with online software JASPAR. (B) ChIP assay was conducted to verify that KLF9 may bind CYP1B1 promoter, and the enrichment of CYP1B1 promoter in the immunoprecipitated DNA‐protein complex was determined with PCR. (C) KLF9 increased CYP1B1 promoter luciferase activity. KLF9 shRNA and pcDNA‐CYP1B1 were individually or altogether transfected into chondrocytes, and treated with 10 ng/mL of IL‐1β for 24 h. (D–H) The mRNA levels of KLF9 and CYP1B1 were detected by qPCR and the protein levels of KLF9 and CYP1B1 were detected by Western blotting. (I) Cell viability was analysed by CCK8 assay. (J) Cell death was analysed with PI staining. (K, L) TNF‐α and IL‐6 secretion levels were detected by ELISA kits. (M) The protein expression of collagen II, Aggrecan, MMP13 and ADAMTS‐5 in cells were analysed by Western blotting. All data are shown as means ± SEM. *N* = 6. One way analysis of variance (ANOVA) followed by Tukey HSD test were applied for evaluating the significance among multiple groups. **p < 0.01.

CYP1B1 is a key gene in inducing ferroptosis in cells, and a previous study has shown that gestational exposure to 1‐NP induced ferroptosis in placental trophoblasts via CYP1B1/ERK signalling pathway [23]. In addition, a previous study showed that KLF9 might be involved in the transcription of CYP1B1 [24]. The results of JAPAR online revealed that −1856 to −1844 and −476 to −464 on the CYP1B1 promoter were KLF9 binding sites (Figure 5A). The results of Ch‐IP assay showed that KLF9 was enriched in the CYP1B1 promoter region (Figure 5B). Luciferase reporter gene assay indicated that KLF9 dramatically increased CYP1B1 promoter activity with an increase in binding sites (Figure 5C). Furthermore, KLF9 shRNA and pcDNA‐CYP1B1 were individually or altogether transfected into chondrocytes. The results of qPCR and Western blotting showed that KLF9 shRNA suppressed KLF9 mRNA and protein expression, and KLF9 levels remained unchanged when CYP1B1 was overexpressed (Figure 5D,E). And KLF9 shRNA inhibited CYP1B1 mRNA and protein expression, whereas overexpression of CYP1B1 restored the repression of KLF9 shRNA on CYP1B1 expression (Figure 5F–H). CCK8 results suggested that pcDNA‐CYP1B1 reversed the promoting effect of KLF9 silencing on the cell viability in IL‐1β‐incubated chondrocytes (Figure 5I). The PI results showed that pcDNA‐CYP1B1 reversed the inhibitory effect of KLF9 silencing on cell death in IL‐1β‐incubated primary chondrocytes (Figure 5J). Moreover, in comparison to treatment with IL‐1β alone, knockdown of KLF9 inhibited the secretion levels of TNF‐α (Figure 5K) and IL‐6 (Figure 5L), while increased the protein expression of Collagen II (Figure 5M and Figure S2A) and Aggrecan (Figure 5M and Figure S2B). However, pcDNA‐CYP1B1 negated these effects. (Figure 5M). Meanwhile, pcDNA‐CYP1B1 also restored the inhibitory effect of KLF9 shRNA on the protein expression of MMP13 and ADAMTSA‐5 (Figure 5M and Figure S2C,D). Based on these results, we considered that knockdown of KLF9 leads to the downregulation of CYP1B1 expression in OA.

### 3.6 CYP1B1 Promoted Chondrocyte Ferroptosis by Regulating ERK

**FIGURE 6:**
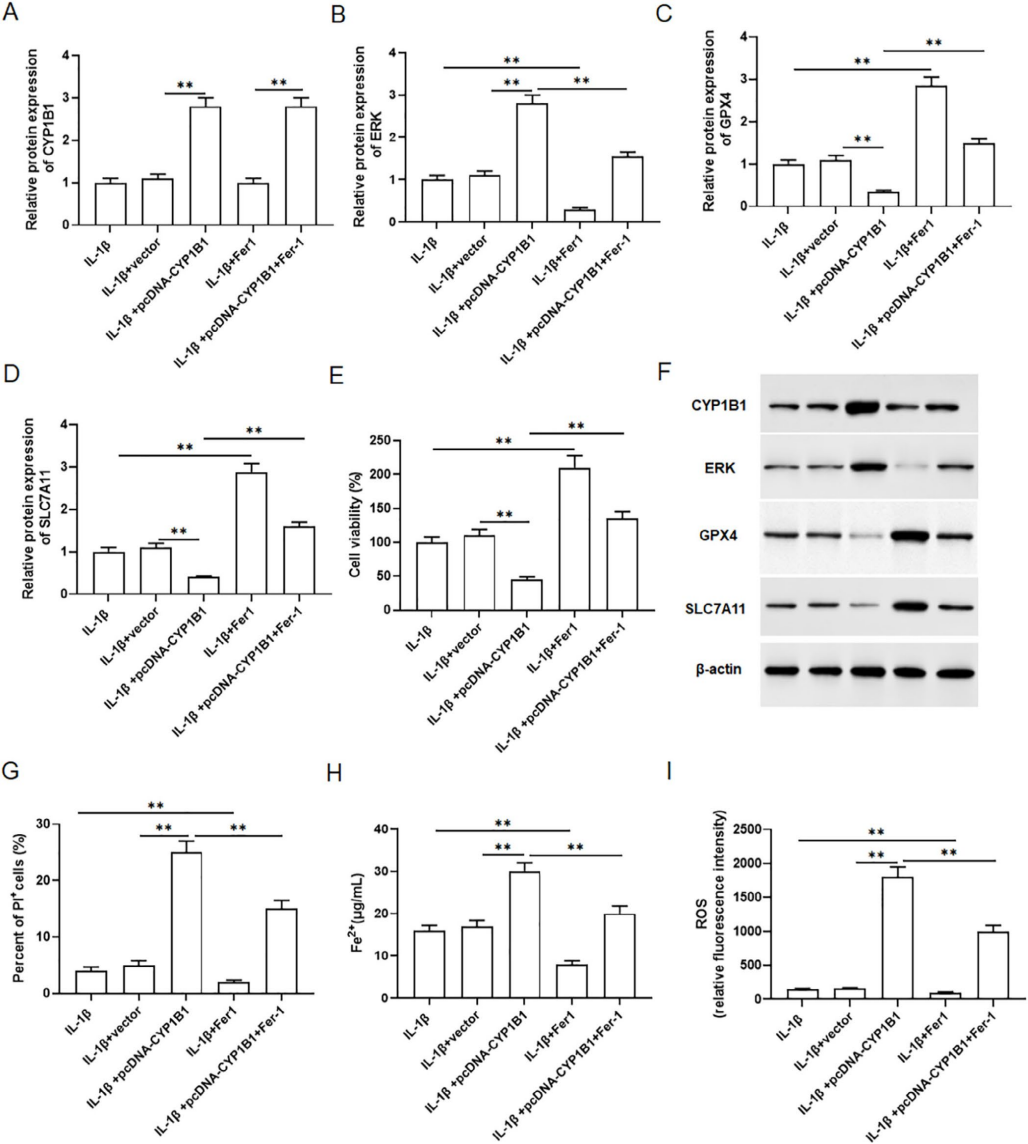
CYP1B1 promoted chondrocyte ferroptosis by regulating ERK. Primary chondrocytes were transfected with pcDNA‐CYP1B1, treated with 10 ng/mL IL‐1β for 24 h and incubated with 1 μmol/L Fer‐1. (A) The protein expression of CYP1B1 was detected by Western blotting. (B) The protein level of ERK was detected by Western blotting. (C, D) The protein levels of GPX4 and SLC7A11 were detected by Western blotting. (E) Cell viability was analysed by CCK8 assay. (F) Western blotting protein band diagram. (G) Cell death was analysed with PI staining. (H, I) The levels of Fe^2+^ and ROS were performed by kits. All data are shown as means ± SEM. *N* = 6. One way analysis of variance (ANOVA) followed by Tukey HSD test were applied for evaluating the significance among multiple groups. **p < 0.01.

The primary chondrocytes were transfected with pcDNA‐CYP1B1 and treated with 10 ng/mL of IL‐1β for 24 h, and then incubated with 1 μmol/L ferrostain‐1 (Fer‐1). Compared with the IL‐1β group, overexpression of CYP1B1 promoted CYP1B1 protein expression and Fer‐1 did not affect CYP1B1 expression in IL‐1β treated chondrocytes, indicating that transfection was successful (Figure 6A,F). Compared with IL‐1β vector group, overexpression of CYP1B1 promoted the expression of ERK, inhibited the expression of GPX4 and SLC7A11, but these changes were abolished by Fer‐1 treatment (Figure 6B–D,F). Moreover, CCK8 assay indicated that overexpression of CYP1B1 decreased the viability of IL‐1β treated chondrocytes, and cell viability was restored with the intervention of Fer‐1 (Figure 6E). PI staining analysis showed that overexpression of CYP1B1 promoted IL‐1β treated chondrocyte death, and was restored by the intervention of Fer‐1 (Figure 6G). Interestingly, Fe^2+^ content (Figure 6H) and ROS levels (Figure 6I) were increased after overexpression of CYP1B1, but this change was restored by the intervention of Fer‐1.

### 3.7 Knockdown of KLF9 Alleviated the Progression of Osteoarthritis in Rats

**FIGURE 7:**
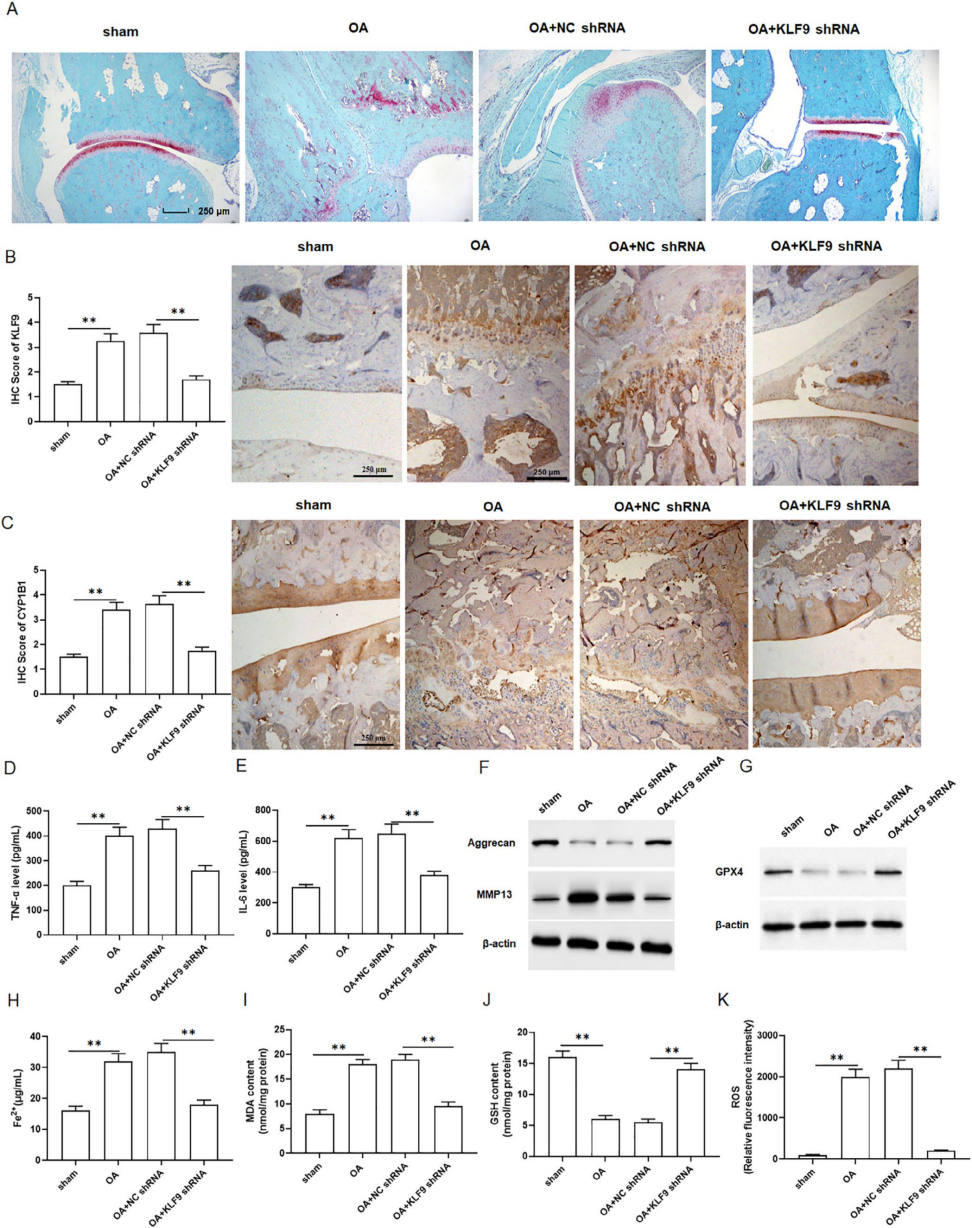
Knockdown of KLF9 alleviated the progression of osteoarthritis in rats. Forty rats were randomly divided into four groups (*n* = 10 per group): The sham group, OA group, OA + NC shRNA group and the OA + KLF9 shRNA group. KLF9 shRNA lentiviral vector injected into rat knee joints (A) Representative images of safranin O/fast green staining of knee joint cartilage tissues. (B, C) Immunohistochemical results of KLF9 and CYP1B1. (D, E) TNF‐α and IL‐6 secretion levels were detected by ELISA kits. (F) The protein expression of Aggrecan and MMP13 were detected by Western blotting. (G) Protein level of GPX4 was detected with Western blotting. (H) The content of Fe^2+^ was detected by using kits. (I–K) The contents of ROS, MDA and GSH were detected with kits. All data are shown as means ± SEM. One way analysis of variance (ANOVA) followed by Tukey HSD test were applied for evaluating the significance among multiple groups. **p < 0.01.

Safranin O/fast green staining results indicated that downregulation of KLF9 ameliorated cartilage tissue damage in OA rats (Figure 7A). IHC results revealed that the protein expression of KLF9 and CYP1B1 were increased in OA compared to the sham group, whereas KLF9 and CYP1B1 were decreased by knockdown of KLF9 (Figure 7B,C). We observed a significant increase in serum levels of inflammatory cytokines TNF‐α and IL‐6 in OA rats compared to the sham group, while their levels were decreased by knockdown of KLF9 (Figure 7D,E). Moreover, the protein expression of Aggrecan was decreased, and the protein expression of MMP13 was increased in the cartilage tissues of OA rats, whereas these changes were abolished by knockdown of KLF9 (Figure 7F and Figure S3A,B). Western blotting demonstrated that the protein expression of GPX4 (Figure 7G and Figure S3C) decreased in the cartilage tissues of OA rats, and restored with the knockdown of KLF9. In addition, the contents of Fe^2+^ (Figure 7H), MDA (Figure 7I) and ROS (Figure 7J) were increased, while GSH content (Figure 7K) was decreased in the joint fluid of OA rats, but these changes were restored by knockdown of KLF9. Altogether, these results demonstrated that knockdown of KLF9 alleviated OA progression in rats.

## 4 Discussion

In the current study, we probed into a novel regulator KLF9 in OA. KLF9 was highly expressed in OA cartilage tissues and IL‐1β‐incubated chondrocytes. Overexpression of KLF9 promoted ERK‐mediated ferroptosis of osteoarthritic chondrocytes through transcriptionally regulating CYP1B1 expression, leading to OA progression. These findings were expected to provide a new theoretical and experimental basis for the pathogenesis and therapeutic targets of OA.

KLF9 is involved in the regulation of many important biological processes, including proliferation, apoptosis, differentiation and development [25]. Recent studies showed that KLF9 was related to cellular oxidative stress damage [26, 27]. Li et al. demonstrated that overexpression of KLF9 might result in oxidative stress damage, mitochondrial apoptosis, and impairment of cellular biological functions in HTR8/Svneo cells, as evidenced by significantly increased levels of MDA and ROS, along with decreased levels of SOD and GSH [28]. Yan et al. indicated that KLF9 exacerbated ischemic damage in myocardial cells by promoting the production of ROS through downregulation of antioxidant thioredoxin reductase 2 (Txnrd2) [26]. ROS contributed to the occurrence of ferroptosis by participating in the formation of lipid peroxides. MDA, GSH and SOD were derivatives of lipid peroxidation and were markers of oxidative stress and ferroptosis [29]. Furthermore, previous research showed that overexpression of KLF9 upregulated ROS levels in macrophage functions, promoting ferroptosis in M1 macrophages [24]. Our study revealed that knockdown of KLF9 inhibited IL‐1β‐induced ferroptosis in chondrocytes. Moreover, previous studies suggested that KLF9 played a vital role in the progression of various bone related diseases such as rheumatoid arthritis and osteosarcoma [25, 30]. Our study revealed that KLF9 overexpression exacerbated OA progression in vitro by promoting chondrocyte apoptosis and increasing the protein expression of cartilage marker and matrix degrading enzymes.

DNMT1 is a type of DNA methyltransferase that inhibits gene transcription by increasing the methylation of gene promoters [31]. Previous research has shown that DNMT1 suppressed ferroptosis by regulating the methylation of ferroptosis‐related genes [32]. DNMT1 suppressed cellular ferroptosis by inducing the methylation of ALOX15 and reducing its expression [33]. In addition, growing evidence suggested that the regulation of DNA methylation by DNMTs played a significant role in the pathogenesis of OA. Ren et al. found that silencing LASP1 inhibited methylation of the TJP2 promoter region through DNMT1 interaction, thereby reducing joint cartilage damage in OA mice [34]. Lv et al. showed that GSK3B recruited DNMT1 to the NR4A3 promoter region to promote NR4A3 methylation, thereby promoting the methylation of NR4A3, inhibiting its expression and mitigating cartilage degradation in OA [35]. In the present study, we found that DNMT1 expression was downregulated in IL‐1β‐incubated chondrocytes. Furthermore, overexpression of DNMT1 was found to inhibit KLF9 expression and alleviate OA progression.

CYP1B1 has been identified as a significant regulatory factor in the process of ferroptosis, as it induces mitochondrial dysfunction and leads to excessive production of ROS [36]. A study demonstrated that AKR1C1 inhibited ferroptosis in endothelial cell carcinoma (ECC) cells by reducing the expression of CYP1B1 [37]. It is well known that the ERK signalling pathway induced ferroptosis in various diseases [38]. Kim et al. indicated that cannabidiol induced ERK activation to promote ferroptosis in glioblastoma cells [39]. Liu et al. suggested that spermidine/spermine N1‐acetyltransferase 1 (Sat1) activated ferroptosis through the MAPK/ERK pathway to promote myocardial ischemia–reperfusion injury [40]. Furthermore, research confirmed that overexpression of CYP1B1 induced ferroptosis by activating the ERK signalling pathway [23]. Consistent with our findings, we indicated that overexpression of CYP1B1 promoted ferroptosis in chondrocytes via the ERK pathway, thereby contributing to the progression of OA. This also accords with earlier study, which showed that CYP1B1 promoted mesenchymal stem cell senescence and led to the progression of OA [36].

Ferroptosis is a specialised form of cellular necrosis that differs morphologically, biochemically and genetically from conventional death as an iron‐dependent lipid peroxidation process. Ferroptosis occurs in subregions of the endoplasmic reticulum, mitochondria associated membranes and mitochondrial inner membranes, and plays a key role in the occurrence and development of various diseases [41, 42, 43]. Recently, Yao et al. demonstrated that chondrocyte ferroptosis contributed to OA progression [44]. Therefore, comprehensive investigations on the mechanism of ferroptosis and the molecular mechanisms that regulate chondrocyte ferroptosis are expected to provide new targets and a therapeutic basis for the clinical treatment of OA. Previous studies showed that D‐mannose reduced the progression of OA by inhibiting ferroptosis of chondrocytes in a HIF‐2α‐dependent manner [45], and Fork‐head box O3 attenuated OA by suppressing ferroptosis through inactivation of NF‐KB/MAPK signalling [46]. Consistent with previous findings, our results demonstrated that KLF9 promoted ERK‐mediated ferroptosis of osteoarthritic chondrocytes through transcriptionally regulating CYP1B1.

In summary, our study demonstrated that KLF9 level was upregulated in IL‐1β stimulated primary chondrocytes. The expression of KLF9 was regulated by DNA methylation of DNMT1, overexpression of KLF9 promoted ERK‐mediated ferroptosis of osteoarthritic chondrocytes through transcriptionally regulating CYP1B1, and knockdown of KLF9 inhibited ferroptosis and reduced cartilage damage in OA rats. Our findings provide a new insight into the pathogenesis of OA and a theoretical basis for the development of KLF9 as a potential therapeutic target for OA.

## Author Contributions

**Min Lv:** conceptualization (equal), investigation (lead), supervision (equal), writing – original draft (equal). **Yuanzhen Cai:** methodology (equal), validation (equal), writing – original draft (equal). **Weikun Hou:** methodology (equal), resources (equal), validation (equal). **Kan Peng:** methodology (equal), resources (equal), validation (equal). **Ke Xu:** methodology (equal), resources (equal), validation (equal). **Chao Lu:** data curation (equal), formal analysis (equal), software (equal), visualization (equal). **Wenxing Yu:** data curation (equal), formal analysis (equal), software (equal), visualization (equal). **Weisong Zhang:** resources (equal), software (equal), supervision (equal), visualization (equal). **Lin Liu:** conceptualization (equal), methodology (lead), project administration (lead), resources (equal), supervision (equal), writing – review and editing (equal).

## Ethics Statement

This study was approved by the Ethics Committee of Xi'an Jiaotong University (NO. XJTULAC‐2023‐116).

## Consent

All authors consent to publish this work.

## Conflicts of Interest

The authors declare no conflicts of interest.

## Supporting information


Data S1.

## Data Availability

The datasets generated during the current study are available from the corresponding author on reasonable request.
