# Supplementary material for: KLF9, Epigenetic Silenced by DNMT1, Promotes ERK‐Mediated Ferroptosis of Osteoarthritic Chondrocytes Through Transcriptionally Regulating CYP1B1
Source: J Cell Mol Med. 2025 Feb 27;29(5):e70375. doi: 10.1111/jcmm.70375 (PMC11867933; doi:10.1111/jcmm.70375)
Supplement: Supplementary file 1 — Data S1. [file JCMM-29-e70375-s001.docx]

**Supplementary Figure legends**


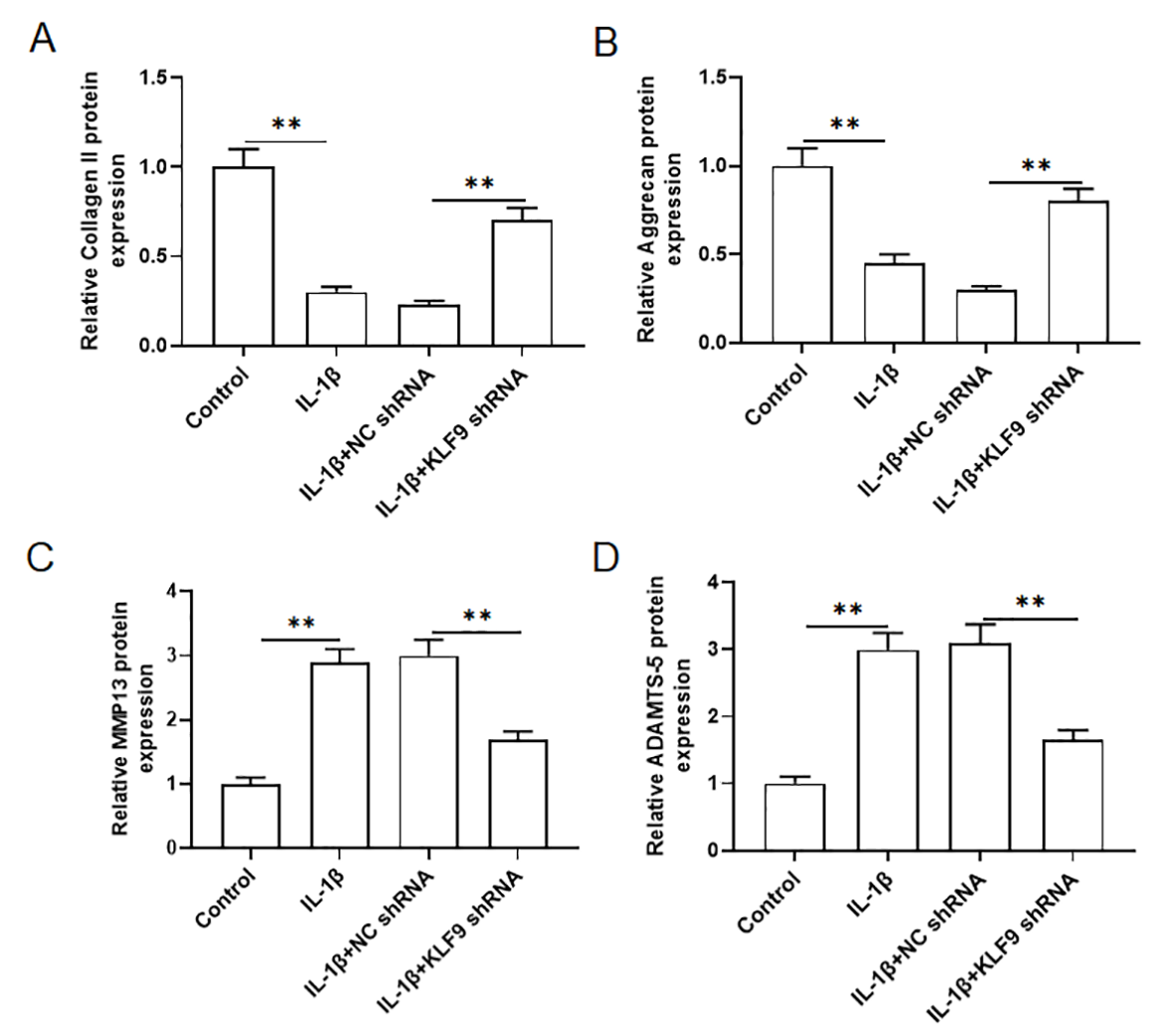


**Supplementary Figure 1** The protein expression of collagen II (A), Aggrecan (B), MMP13 (C) and ADAMTS-5 (D) in chondrocytes were analyzed by Western blotting. All data are shown as means ± SEM. N = 6. One way analysis of variance (ANOVA) followed by Tukey HSD test were applied for evaluating the significance among multiple groups. ***P* < 0.01.


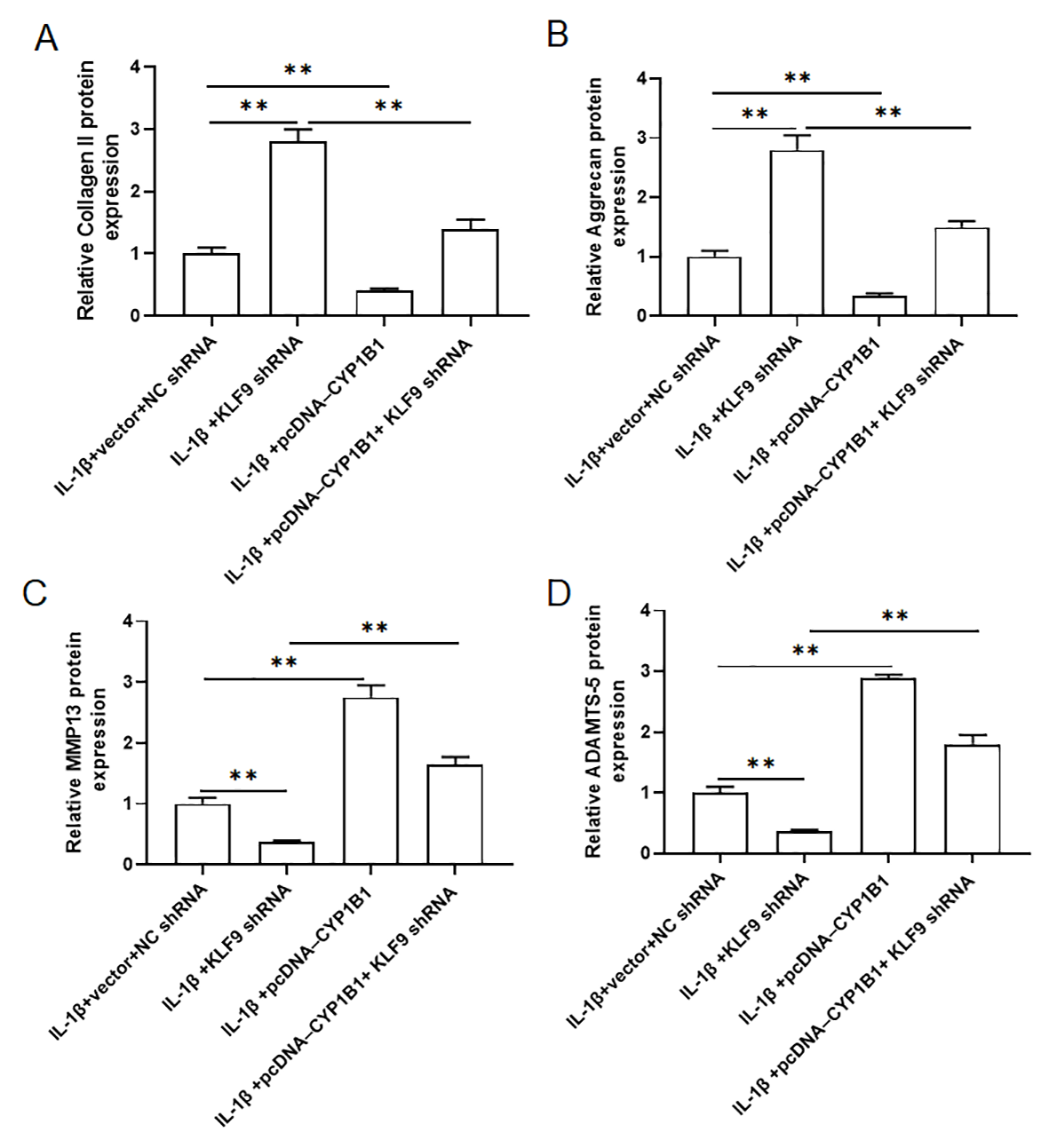


**Supplementary Figure 2** The protein expression of collagen II (A), Aggrecan (B), MMP13 (C) and ADAMTS-5 (D) in chondrocytes were analyzed by Western blotting. One way analysis of variance (ANOVA) followed by Tukey HSD test were applied for evaluating the significance among multiple groups. ***P* < 0.01.


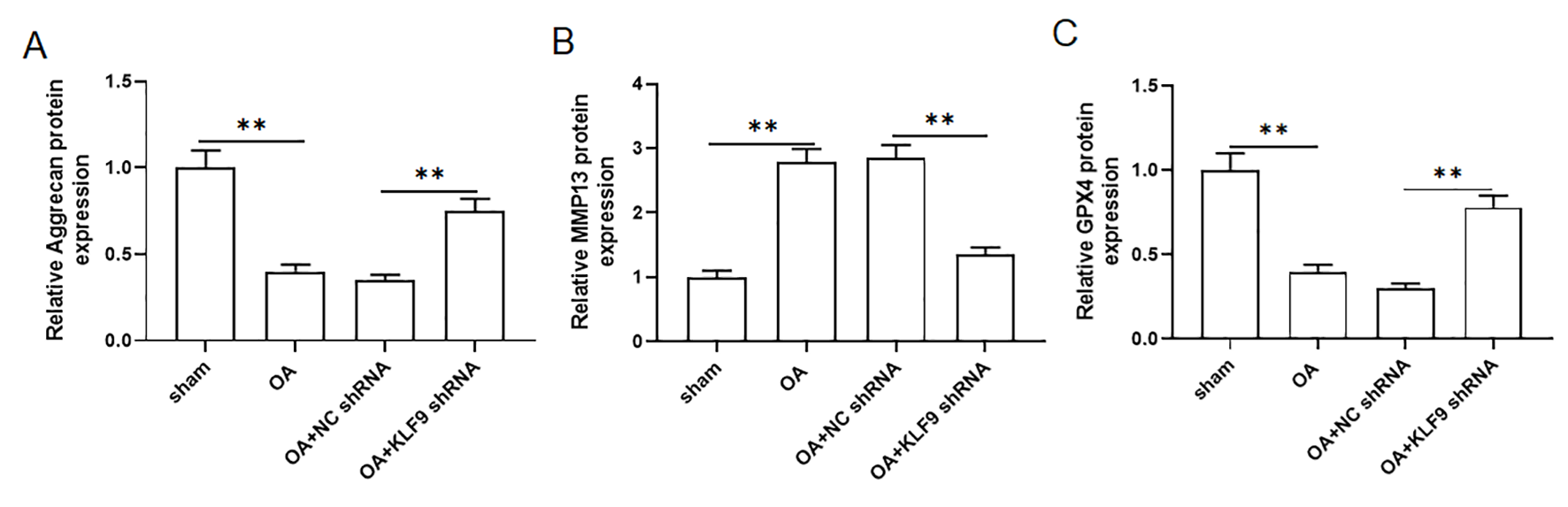


**Supplementary Figure 3** The protein expression of Aggrecan (A) and MMP13(B) were detected by Western blotting. Protein level of GPX4 (C) was detected with Western blotting. One way analysis of variance (ANOVA) followed by Tukey HSD test were applied for evaluating the significance among multiple groups. ***P* < 0.01.
